# Factors associated with loss to follow-up among women in Option B+ PMTCT programme in northeast Ethiopia: a retrospective cohort study

**DOI:** 10.7448/IAS.19.1.20662

**Published:** 2016-03-21

**Authors:** Israel Mitiku, Mastewal Arefayne, Yonatal Mesfin, Muluken Gizaw

**Affiliations:** 1 Department of Public Health, College of Medicine and Health Sciences, Wollo University, Dessie, Ethiopia; 2 Department of Preventive Medicine, School of Public Health, College of Health Sciences, Addis Ababa University, Addis Ababa, Ethiopia

**Keywords:** loss to follow-up, Option B+, Ethiopia

## Abstract

**Introduction:**

Ethiopia has recently adopted lifelong antiretroviral therapy (ART) for all HIV-positive pregnant and breastfeeding women (Option B+ strategy), regardless of CD4 count or clinical stage. However, the exact timing and predictors of loss to follow-up (LFU) are unknown. Thus, we examined the levels and determinants of LFU under Option B+ among pregnant and breastfeeding women initiated on lifelong ART for prevention of mother-to-child transmission (PMTCT) in Ethiopia.

**Methods:**

We conducted a retrospective cohort study among 346 pregnant and breastfeeding women who started ART at 14 public health facilities in northeast Ethiopia from March 2013 to April 2015. We defined LFU as 90 days since the last clinic visit among those not known to have died or transferred out. We used Kaplan-Meier and Cox proportional hazards regression to estimate cumulative LFU and identify the predictors of LFU, respectively.

**Results:**

Of the 346 women included, 88.4% were pregnant and the median follow-up was 13.7 months. Overall, 57 (16.5%) women were LFU. The cumulative proportions of LFU at 6, 12 and 24 months were 11.9, 15.7 and 22.6%, respectively. The risk of LFU was higher in younger women (adjusted hazard ratio (aHR) 18 to 24 years/30 to 40 years: 2.3; 95% confidence interval (CI): 1.2 to 4.5), in those attending hospitals compared to those attending health centres (aHR: 1.8; 95% CI: 1.1 to 3.2), in patients starting ART on the same day of diagnosis (aHR: 1.85; 95% CI: 1.1 to 3.2) and missing CD4 cell counts at ART initiation (aHR: 2.3; 95% CI: 1.2 to 4.4).

**Conclusions:**

The level of LFU we found in this study is comparable with previous findings from other resource-limited settings. However, high early LFU shortly after ART initiation is still a major problem. LFU was high among younger women, those initiating ART on the day of HIV diagnosis, those missing baseline CD4 count and those attending hospitals. Thus, targeted HIV care and treatment programmes for these patients should be part of future interventions to improve retention in care under the Option B+ PMTCT programme.

## Introduction

In 2013, an estimated 3.2 million children under the age of 15 were living with HIV globally, 91% of whom were in sub-Saharan Africa [1]. Mother-to-child transmission (MTCT) of HIV remains the primary mode of infection in children [2]. Timely access to quality life-saving antiretroviral (ARV) drugs during and after pregnancy is a proven intervention to preserve maternal health and virtually eliminate the risk of MTCT of HIV [2,3].

In 2010, the World Health Organization (WHO) published prevention of mother-to-child transmission (PMTCT) guidelines. The guidelines specify that a CD4 count is crucial to decisions on the eligibility of HIV-positive pregnant women for lifelong antiretroviral therapy (ART) [4]. In 2013, the WHO issued a new guideline recommending lifelong ART for all HIV-positive pregnant and breastfeeding women, regardless of their CD4 count or WHO clinical stage (Option B+) [5]. The rationale of this policy was to achieve the elimination of new paediatric HIV infections by 2015 and ensure that all ART-eligible pregnant women receive triple ARVs for their own health [5,6]. This approach is believed to contribute to the target of ending the AIDS epidemic by 2030 [7].

Option B+ may be a more effective PMTCT strategy, as it can help overcome some barriers (like poor access to CD4 testing) associated with achieving high coverage of treatment [8]. This approach ensures that most HIV-positive women are placed on treatment immediately following diagnosis, which leads to further reduction of MTCT [8]. Moreover, the simplification of drug regimen options could make adherence easier for both mother and healthcare provider, which is likely to facilitate higher retention rates [9,10]. Additionally, Option B+ provides an excellent opportunity to begin roll-out of “treatment as prevention,” which can have a significant impact in reducing new HIV infections due to sexual transmission among serodiscordant partners [9].

The implementation of Option B+ has already begun to show impressive results in resource-constrained settings dramatically increasing the numbers of pregnant and breastfeeding women enrolled on ART [9]. The gains sought by expanding ART access through Option B+ depend on the proportion of women who adhere to treatment and are retained in care. However, these gains in access are being challenged by high loss to follow-up (LFU) and non-adherence to treatment that significantly undermines success of Option B+ [11,12]. As an illustration, in Malawi, 17% of women in Option B+ are lost to follow-up six months after ART initiation [13]. Poor retention in care or adherence to treatment could be a threat to both HIV-positive women and their infants. Retention in HIV care is one of the crucial indicators of the success of ART programmes [14,15]. Poor retention due to LFU is associated with virological failure, development of potent drug-resistant virus as well as maternal HIV disease progression and increased risk of MTCT [16,17].

One of the challenges when analyzing the potential effectiveness of PMTCT interventions is LFU of mothers [18,19]. Several reasons may explain why women starting ART do not attend their follow-up appointments. Some qualitative studies have identified factors contributing to LFU under the Option B+ programme including lack of motivation to adhere to lifelong medication after a healthy delivery [18,19], denial or lack of disclosure of HIV status [20] and not feeling ready to embark on lifelong medication [19]. In addition, high LFU was reported among younger women and women who initiated ART during pregnancy [21].

In 2013, Ethiopia launched Option B+ as a national policy to prevent MTCT. This ART policy was implemented in a phased approach, prioritizing health centres that provide both PMTCT and ART services [22]. To date, with the exception of one adherence study [23], there are no published studies concentrating on LFU under this new modality in Ethiopia. Further examination of LFU and its risk factors among women starting ART under the Option B+ strategy is warranted.

The objectives of this analysis were to determine the levels, timings and determinants of LFU under Option B+ among pregnant and breastfeeding women initiating lifelong ART at 14 public health facilities in northeast Ethiopia. The findings of this study will assist in developing evidence-based interventions to promote retention in care, such that the health benefits of Option B+ can be fully realized.

## Methods

### Ethiopian national PMTCT programme

HIV-positive pregnant and breastfeeding women start on lifelong ART (fixed-dose combination of tenofovir/lamivudine/efavirenz) regardless of their immunological status in accordance with the Option B+ strategy stipulated in Ethiopia's comprehensive guidelines for PMTCT/maternal, neonatal and child health (MNCH) [22]. All pregnant women, except those with advanced HIV disease (WHO Stage 3 and 4) and opportunistic infections (OIs), start on ART during antenatal care. Pregnant women with advanced disease are referred to an ART clinic for diagnosis and treatment of the OI and initiation of ART. They are then transferred back to PMTCT for their ongoing care and treatment. The guideline recommends that tracing of women lost to care be initiated within seven days of a missed appointment.

### Study setting

The study was implemented at public health institutions located in three zones (including South Wollo, North Wollo and the Oromia special zone) of the Amhara region in northeast Ethiopia. We selected 14 facilities based on the numbers of women on ART and accessibility to study staff for data collection. The selected facilities included the following: one referral hospital, three general hospitals and 10 urban health centres. All of the health facilities provide integrated MNCH/PMTCT services to HIV-positive women free of charge. All the facilities used a similar model of PMTCT delivery, in which HIV-positive women were initiated on ART and followed up at the antenatal clinic. Clinic appointments were typically scheduled one to three months apart, based on patient's clinical status.

### Study design and population

We conducted a retrospective cohort study of women who started ART under the new Option B+ treatment guidelines between 8 March 2013 and 13 April 2015. Only patients who enrolled at least three months prior to the end of data collection were eligible to be included in the sample, so that all patients had the opportunity to meet the definition for LFU. Women whose reason for starting ART was unknown were excluded. At each site, patients matching inclusion criteria were identified using MNCH registers covering the study time periods. Medical record numbers were retrieved from this registry and used to find each individual's patient card.

### Study variables and data collection

In this study, the explanatory variables were baseline demographic characteristics and clinical variables including age at ART initiation, marital status, education, religion, place of residence (urban/rural), baseline weight, pregnancy status (pregnant/breastfeeding), time between diagnosis and ART initiation (started ART same day of HIV diagnosis/started ART later), CD4 cell count at ART initiation, WHO clinical stage (Stage 1/2 vs. 3) and year of ART initiation. Health facility type (hospital vs. health centre) was also included. Baseline CD4+ cell counts were taken as the CD4+ cell count nearest to ART initiation, using a window of three months before and one month after the date of ART initiation. For most patients, data on CD4+ cell count were missing. We therefore created dummy variables that indicated whether or not CD4 cells had been assessed within a window period of three months prior to and one month after ART initiation.

The data sources were the Federal Ministry of Health patient card, ART intake forms, HIV care follow-up and the PMTCT register. Trained nurses working in the ART clinic/PMTCT extracted the data using a structured tool prepared for the study. The data abstraction tool for chart review was prepared based on the information contained within the patient registration and follow-up card.

### Study outcomes and definitions

The primary outcome of this study was LFU. LFU was defined as 90 days after the last documented visit, as per the recently developed simplified tools to measure retention in care in ART programmes [24]. Time to LFU was a secondary end point of the study. We estimated follow-up time on ART as the time between ART initiation and either documented transfer out, LFU (as defined above) or completion of observation period (13 April 2015), whichever occurred first. For those who never attended a follow-up visit we defined time to LFU as the time from ART initiation to 15 days after the ART initiation. Patients who had documentation of transfer out were censored at the date of transfer or last visit date, whichever was later.

### Data analysis

EpiData version 3.1 was used for data entry and STATA version 11 (StataCorp, College Station, TX, USA) for statistical analyses. We used medians and interquartile ranges (IQRs) for continuous variables and proportions for categorical variables to describe baseline characteristics. Proportions LFU at 6, 12 and 24 months after ART initiation were estimated using Kaplan-Meir methods. Cox proportional hazards regression models with robust sandwich estimates to account for within-facility correlations were used to examine the relationships of individual and facility-related independent variables with the risk of LFU. We included all the variables associated with LFU in the univariate analysis (at the *p*<0.20 level) in the multivariable Cox regression model. Associations were estimated using hazard ratios with 95% confidence intervals (CI). Finally, associations were examined at a significance level of *p*<0.05 (two-sided test). The proportional hazards assumption was checked by graphical methods and using Schoenfeld residuals tests and the results of these analyses suggested that the proportional hazards assumption appeared to be reasonable. We used both the Akaike and Bayesian information criteria (AIC/BIC) to assess model parsimony.

### Ethical consideration

Ethical approval for this study was granted by the Research Ethics Committee of Wollo University and exemption was given by the respective local authority where the study was implemented. The study utilized data that are routinely collected for service delivery and anonymized for analysis; thus we did not seek patient consent.

## Results

**Figure 1:**
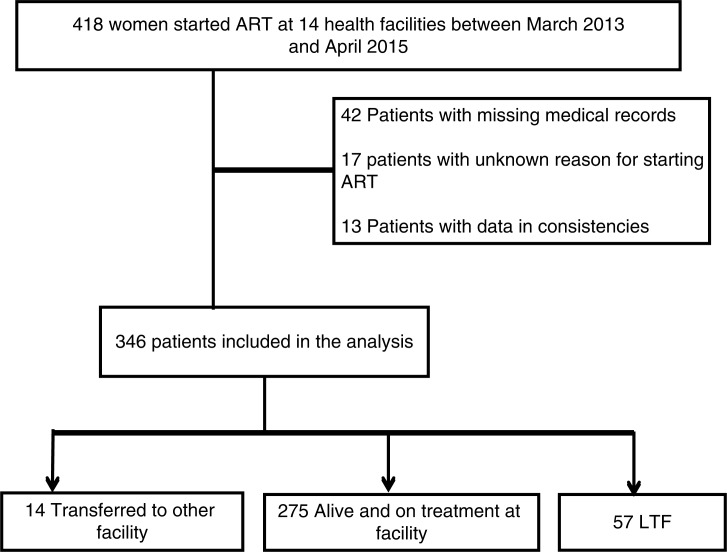
CONSORT diagram with total number of eligible patients and number of patients included in the analysis.

Between 8 March 2013 and 13 April 2015, a total of 418 HIV-positive women started ART under Option B+ at one of the 14 public health facilities included in the study. Of these, 42 were excluded from the study because their charts could not be found. Of the 376 remaining women, 30 were excluded because their reason for starting ART was unknown (*n*=17) or due to data inconsistencies (*n*=13), resulting in a final total of 346 women for further analysis (Figure 1). About two-thirds of the patients (67.3%) were enrolled at public health centres; the remaining 15.9 and 16.8% started ART at referral and general hospitals, respectively.

### Patient characteristics at ART initiation

The demographic characteristics of the study population at ART initiation are shown in Table 1. The median (IQR) age at ART initiation was 26 (23 to 30) years. A majority of women (80.3%) were married, and 88.7% were urban residents. Nearly half (51.2%) were Orthodox Christian and 45.7% were Muslims. Most patients received either no formal education (30.3%) or education only up to the primary level (36.1%); 4.3% had incomplete information on level of education. The median (IQR) weight at ART initiation was 55 (50 to 61) kg and more than half (55.5%) of patients had body weight less than 55 kg. Overall, 89.7% (289) of women were pregnant and the median (IQR) gestational age at ART initiation was 20 weeks (IQR: 15 to 26).

**Table 1:** Baseline demographic and clinical characteristics of women starting ART under Option B+ from March 2013 to April 2015, Ethiopia

	Total		Women who were LFU (*n=*57)		Women who were not LFU (*n=*289)		
				
Characteristics	*n*	%	*n*	%	*n*	%	*p*
Age at ART initiation							
18 to 24	121	35.0	33	57.9	88	30.4	0.042
25 to 29	138	39.9	12	21.1	126	43.6	0.190
30 to 40	87	25.1	12	21.1	75	26.0	
Place of residence							
Urban	307	88.7	49	86.0	258	89.3	
Rural	39	11.3	8	14.0	31	10.7	0.352
Marital status							
Married	290	83.8	45	78.9	245	84.8	
Single	26	7.5	5	8.8	21	7.3	0.454
Divorced/widower	30	8.7	7	12.3	23	7.9	0.374
Religion							
Orthodox	177	52.4	34	59.6	143	50.9	
Muslim	158	49.7	23	40.4	135	48.0	
Protestant	3	0.9	0	0.0	3	1.1	
Missing	8						
Educational status							
No education	105	31.7	17	31.5	88	31.8	
Primary	125	37.8	23	42.6	102	36.8	0.563
Secondary	84	25.4	10	26.7	74	26.7	0.414
Tertiary	17	5.1	4	7.4	13	4.7	0.614
Missing	15						
Pregnancy status at enrolment							
Pregnant	306	88.4	53	93.0	253	87.5	
Breastfeeding	40	11.6	4	7.0	36	12.5	0.255
Weight at ART initiation (kg)							
≤55	134	38.7	27	47.4	165	57.7	0.168
>55	212	61.3	30	52.6	121	42.3	
Missing							
WHO clinical stage at ART initiation							
Stage 1/2			54	94.7	270	93.4	0.894
Stage 3			3	5.3	19	6.6	
Facility level							
Health centre	232	67.1	31	54.4	201	69.5	
Hospital	114	32.9	26	45.6	88	30.5	0.036
Started ART on the day of diagnosis							
No	196	57.1	23	40.4	173	60.5	<0.001
Yes	147	42.9	34	59.6	113	39.5	
Missing	3						
Year of ART initiation							
2015	56	16.2	5	8.8	51	17.6	
2014	185	53.5	35	61.4	150	51.9	0.865
2013	105	30.3	17	29.8	88	30.4	0.331
CD4 cell count done near (3 months before and 1 month after) ART initiation							
Yes	324	93.6	13	22.8	121	41.9	
No	22	6.4	44	77.2	168	58.1	0.004

ART, antiretroviral therapy; LFU, lost to follow-up.

Overall, 234 (67.9%) women had at least one recorded CD4 cell count. Of these, 134 (57.3%) had a CD4 cell count within a window of three months prior to and one month after ART initiation (three were missing a date for CD4 cell count). The median CD4 was 460 (IQR: 277 to 638) cells/mL. The majority (70.5%) were classified as WHO Clinical Stage 1, followed by those classified as Stage 2 (23.1%) and Stage 3 (6.6%). None of the patients were classified as WHO Clinical Stage 4. Of the patients with a known HIV-testing date, 147 (42.5%) began ART on the day they were diagnosed (three were missing HIV-testing date). The median (IQR) time from diagnosis to ART initiation was 3 (0 to 180) days.

### Loss to follow-up

**Figure 2:**
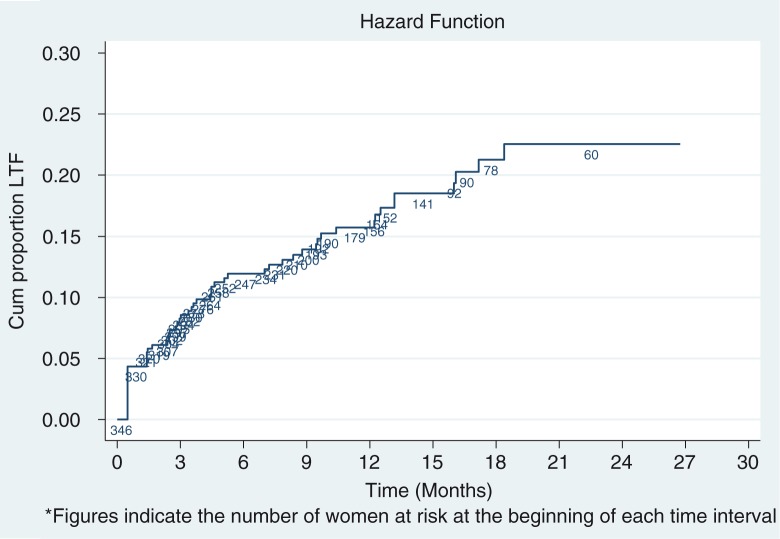
Kaplan-Meier estimates of loss to follow-up among women starting antiretroviral therapy under Option B+ from March 2013 to April 2015, Ethiopia.

Overall, 57 (16.5%) of women were LFU during the observation period. Of the 57 women LFU, 16 (28.0%) had no follow-up visit after the initiation of ART. The cumulative proportion of patients LFU at 6, 12 and 24 months after ART initiation was 11.9% (95% CI: 8.9, 16.0%), 15.7% (95% CI: 12.0, 20.4%) and 22.5% (95% CI: 17.3, 29.2%), respectively (Figure 2).

The overall incidence of LFU among all women, including those who attended no follow-up visit, was 14.8 per 1000 person-years (PY) of observation time (95% CI: 11.4 to 19.2) with a median follow-up of 10.8 months (IQR: 4.5 to 16.7). The LFU rate was high during the first six months of ART initiation (29.1/1000 PY) but fell with increasing time in follow-up, with LFU rates of 14.0/1000 PY and 7.5/1000 PY at 12 and 24 months, respectively.

### Factors associated with LFU

In bivariate analysis, LFU differed significantly from non-LFU in terms of age at ART initiation, baseline weight, facility level, ART initiation from the day of diagnosis and knowledge of baseline CD4 cell count (Table 2).

**Table 2:** Baseline demographic and clinical characteristics associated with LFU among women who started ART under Option B+ from March 2013 to April 2015, Ethiopia

Characteristics	Unadjusted HR (95% CI)	Adjusted HR (95% CI)	*p*
Age at ART initiation			
18 to 24	1.98 (1.1 to 3.8)	2.3 (1.2 to 4.5)	**0.017**
25 to 29	0.59 (0.26 to 1.30)	0.67 (0.3 to 1.5)	0.325
30 to 40	1.00	1.00	
Weight at enrolment (kg)			
≤ 55	1.00	1.00	
> 55	1.42 (0.86 to 2.41)	1.3 (0.8 to 2.3)	0.348
Facility level			
Health centre	1.00	1.00	
Hospital	1.75 (1.04 to 2.96)	1.8 (1.1 to 3.2)	**0.039**
Started ART on the day of diagnosis			
No	1.00	1.00	
Yes	2.56 (1.51 to 4.32)	1.9 (1.1 to 3.2)	**0.032**
CD4 cell count done near (3 months before and 1 month after) ART initiation			
Yes	1.00	1.00	
No	2.47 (1.34 to 4.56)	2.3 (1.2 to 4.4)	**0.010**

*Note:* Multivariable analysis adjusted for all variables shown in table. Hazard ratios estimated using robust sandwich estimators for variance to account for within-clinic correlation. HR, hazard ratio; CI, confidence interval; ART, antiretroviral therapy. Values in bold are statistically significant at *P*≤0.05.

In adjusted analyses, the independent risk factors for LFU were as follows: younger age at ART initiation, missing CD4 cell count at ART initiation, ART initiation on the same day of diagnosis and starting ART at hospital. In unadjusted analyses, weight at ART initiation was associated with LFU, but this was not significant after controlling for other variables.

## Discussion

This is the first study carried out in Ethiopia to report on the levels and determinants of LFU under universal lifelong ART for pregnant and breastfeeding women (Option B+). Approximately 16% of women in the PMTCT Option B+ programme were LFU. Younger age, missing CD4 count at ART initiation, starting ART on the same day of diagnosis and starting ART at hospital were found to be the risk factors for LFU.

The LFU observed in this study is lower compared to previous study findings from Malawi, in which 17% [13,25] and 22% 11 of all women were lost 6 and 12 months after ART initiation, respectively. However, the fact that the definition of LFU varied in each programme or study complicates the comparison between different settings [26].

About 12% of LFU occurred within the first six months of ART initiation. Early patient losses under Option B+ have also been reported from other African settings [13]. Previous studies from Ethiopia [27–29] and other African countries [30,31] have also documented higher risk of attrition in the first six months of ART among adults in the general population. These findings suggest that the initial time period after a patient initiates ART is the most critical to focus efforts aimed at improving retention [31].

In this study, 28.1% of women who were LFU received ARVs only once and never returned for their appointment, suggesting that a proportion of these women never started ART or stopped after the first dose in the health facility. Our finding is lower than a report from Malawi, where 47% of women collected ART at initiation and never returned for their appointment [21]. The difference could be due to differences in the model of PMTCT service delivery and also differences between the study settings [32]. Studies have attributed high dropout rates for Option B+ to resistance to starting lifelong ART. Women may have been less well prepared for ART and thus less likely to attend follow-up visits [19,33]. This idea is supported by our finding of the association between the time between HIV diagnosis and ART initiation and LFU.

Women who started ART on the day they were diagnosed had a higher risk of LFU than women who started ART later. This finding has been documented elsewhere [13]. Previous studies have suggested that women who start ART immediately after diagnosis do not have the chance to disclose their HIV status, and prior disclosure may improve ART adherence [18,34]. Lack of disclosure related to stigma and lack of male partner involvement has also been reported to deter postpartum women in HIV care under Option B+ [18]. Moreover, women's lack of readiness at ART initiation could result in interrupted treatment [35]. Empirical evidence regarding the advantages of systematically delayed ART is limited [36]. Giving sufficient time and information might help clients adjust to the idea of lifetime treatment, but this possibility needs to be better documented.

The other significant factor associated with LFU in our study was women's age at ART initiation. Women who were 18 to 24 years at ART initiation were more likely to be LFU than older women. This finding has been observed by others [21]. Our findings are consistent with the findings reported in the general HIV-positive population accessing ART for personal health [37,38]. Multiple factors may contribute to higher LFU among younger women. One possible explanation for this finding could be lack of belief in the benefits of attending clinic and initiating ART among these women [39]. In contrast, older women may have more settled lifestyles, which allow them to better manage ARVs for PMTCT [21]. Young women might be concerned about starting treatment for life at such a young age [20]. These findings highlight the need to focus efforts toward retaining this group in HIV care.

In our analysis, we found that LFU was likely among women with missing data on CD4 cell count at ART initiation. There are speculations that patient's knowledge of CD4 count could influence initiation and adherence to ART [40]. One of the possible explanations could be that testing CD4 cell count at the first visit raises women's HIV-related literacy and awareness and might engage women in care. The use of point-of-care CD4 count technology has been recommended as one strategy to improve patient retention in pre-ART care [41,42]. In this study, only 38.7% had a CD4 cell count within a window of three months prior to and one month after ART initiation. Although CD4 count is no longer a prerequisite for initiating intervention for HIV-positive pregnant women, the national PMTCT guideline recommends that CD4 count be done as soon as possible as a baseline and for monitoring purposes [22]. The possibility that providing women with their CD4 count at the time of ART initiation might change the attitudes of women toward lifelong care, and thus result in different levels of retention in care, has been recommended as a priority for further operational research [40].

Additionally, LFU was significantly associated with health facility type. A higher hazard of LFU was observed among women attending hospitals compared to those attending primary facilities (health centres). Similar findings were reported by Tenthani *et al*. [13]. from a cohort in Malawi in which LFU was also associated with larger health facilities. Our finding confirms earlier observations of increased risk of attrition among patients attending higher level healthcare facilities [13,43]. It may be related to less favourable health-system factors in larger ART programmes, such as patient burden per staff member and longer waiting times [44]. Misclassification of patients as LFU has also been reported to be more common in larger facilities, as these facilities are located in more urban regions with a mobile population [13].

Being pregnant at ART initiation has been shown to be an independent indicator of LFU among HIV-positive women in sub-Saharan Africa [21]. However, we did not find a similar association in our study, although this could be due to limited sample size.

Though we used patient data collected as part of routine service delivery, the comprehensiveness of patient medical records allowed us to evaluate certain patient-level factors that were previously associated with retention. Availability of HIV-testing date allowed us to describe time elapsed between HIV testing, ART initiation and patient outcomes.

The study has some potential limitations. These limitations were mainly related to data abstraction of routinely collected programme data; hence, we could not measure certain patient-level factors and societal factors. The data for CD4 cell counts for most women were not complete, either because they were not determined or if determined not registered. Another limitation of the study was the use of the pregnant women's weight at ART initiation, which changes continuously and substantially. In addition, patients whose medical record was not found were not included in the analysis, which might have affected the true level of LFU. Finally, we could not independently verify outcomes for those who were classified as LFU. It is possible that some patients may have been classified as LFU due to undocumented transfers or undocumented death.

## Conclusions

In conclusion, the level of LFU we found in this study is similar to previous findings from other resource-limited settings. High early LFU shortly after ART initiation is a major problem. LFU was high among younger women, those initiating ART on the day of HIV diagnosis, those missing baseline CD4 count and those attending larger hospitals. Nonetheless, we recommend additional large-scale research to ascertain to what extent time between HIV diagnosis and ART initiation and knowledge of baseline CD4 count are associated with women's retention in care. Targeted HIV care and treatment programmes for these patient subgroups should be part of future interventions to improve retention in care under Option B+.
